# Privacy-preserving and robust mouse dynamics authentication using hybrid transformer-CNN and federated learning

**DOI:** 10.3389/frai.2026.1914353

**Published:** 2026-09-17

**Authors:** K. Sasikumar, Sivakumar Nagarajan

**Affiliations:** School of Computer Science and Engineering, Vellore Institute of Technology, Vellore, India

**Keywords:** adversarial robustness, behavioral biometrics, continuous verification, federated learning, mouse dynamics

## Abstract

Traditional methods like passwords and PINs are increasingly vulnerable, making continuous authentication essential. Behavioral biometrics such as mouse dynamics provide a non-intrusive way to verify users through their unique interaction patterns. This study proposes a secure and privacy-preserving framework for mouse dynamics authentication using a Hybrid Transformer-CNN model, where the Transformer captures global behavioral dependencies and the Convolutional Neural Network (CNN) extracts local movement patterns. The framework includes a comprehensive preprocessing pipeline with feature engineering, SMOTE-based class balancing, and stratified cross-validation. To enhance security and privacy, the system integrates adversarial robustness evaluation and federated learning with differential privacy, enabling decentralized training without sharing raw user data. The model is further evaluated under non-IID data distributions and open-set authentication scenarios with per-user thresholds to reflect realistic deployment conditions. Experimental results demonstrate high performance, achieving 99.51% accuracy, 99.53% precision, low FPR (0.000256), and EER (0.000068). The model maintains robustness under adversarial attacks and achieves efficient real-time detection. Overall, the proposed framework provides a reliable and privacy-aware solution for continuous authentication in security-sensitive applications.

## 1 Introduction

In today's digital world where everything is connected, it has become necessary to have a very strong user authentication system so that only the authorized users can get access to critical systems, online services, and sensitive data. On the other hand (Siddiqui et al., 2021; Salman and Hameed, 2018), the global average cost of a data breach has reached up to multiple million US dollars per case, and among the most frequent and most expensive are those that are caused by stolen credentials (IBM, 2025). These tendencies are a compelling reason to implement stronger and more continuous authentication systems like behavioral biometrics, which can add an extra layer of security to the conventional password-based ones. The development of cyber threats creates the necessity of strong authentication methods to protect users from identity theft, account takeover, and unauthorized access. The classic ways of authenticating users are largely based on the user's knowledge (passwords, PINs) or possession (tokens, OTPs) of something, and on the other hand, the physiological biometrics like fingerprint, iris, and facial recognition, though providing the strongest initial verification, are limited to one-time authentication at login. After an attacker has compromised valid credentials or has circumvented the first biometric check, there will not be any verification layers to detect misuse in an active session (Deutschmann et al., 2013; Chong et al., 2020).

As a result of this problem, there was an increase in the use of behavioral authentication methods that continuously monitor the user's typical interaction patterns—for instance, keystroke dynamics, touchscreen gestures, and mouse movements—to provide ongoing identity verification. Mouse dynamics, among these methods, has become particularly popular because of its non-invasive approach and the ability to detect individual traits through metrics such as cursor path, speed, acceleration, and click rhythm. By analyzing these subtle and exclusive movement behaviors, mouse dynamics can provide continuous, real-time user authentication, thereby serving as an efficient means of intruder detection and security enhancement in modern digital systems (Mondal and Bours, 2017; Ananya and Singh, 2018). Artificial intelligence methods, especially Deep Learning (DL), are being used more and more to spot and separate the real users and the fake ones by finding out the specific behavioral traits that are hidden in the mouse-movement data. But on the other hand, the training of such models generally requires a lot of behavioral data, which poses a problem in terms of privacy. Federated Learning (FL) is an alternative that preserves privacy, as it permits the model to be developed on the user's device, thus avoiding the transfer of raw mouse data to a central location. Only model updates are shared, and this is how the privacy of users' behavioral patterns is maintained. The combination of DL and FL results in mouse-dynamics authentication that is not only very accurate but also privacy-preserving, thus making it a robust method for permanent and secure user verification (Siddiqui et al., 2022; Antal and Fejér, 2020).

We propose a complete behavioral biometric authentication system in this research that integrates high-level deep learning, security enhancements, and privacy protection techniques. Our approach's primary aim is to enhance the mouse-dynamics-based user authentication in terms of accuracy, strength, and confidentiality. The major technical contributions of this work are as follows:

- A Hybrid Transformer-CNN model is proposed to capture both global behavioral dependencies and local mouse movement patterns, improving authentication accuracy.
- A comprehensive feature engineering and preprocessing framework is developed to extract discriminative mouse dynamics features for reliable user authentication.
- A privacy-preserving federated learning framework is implemented with differential privacy to enable secure decentralized training without sharing raw user data.
- The robustness of the proposed model is evaluated under adversarial attacks (FGSM, PGD, and CW) and GAN-based spoofing scenarios.
- Extensive evaluations are conducted, including open-set authentication with per-user thresholds, non-IID federated learning analysis using Dirichlet distribution, and comparisons with centralized and FL-based baselines.

This study integrates a Hybrid Transformer-CNN model with privacy-preserving federated learning, differential privacy, and adversarial robustness evaluation to address complementary requirements of accuracy, privacy, and security in mouse-dynamics authentication.

## 2 Related work

Mouse dynamics is a very good behavioral biometrics for continuous authentication; it gives non-intrusive and light user verification based on the patterns of the mouse movements (Khan et al., 2024). There has been much research on the use of machine learning and deep learning to increase their accuracy and reliability. We will look at some significant contributions in this field. Siddiqui et al. (2022) have investigated and evaluated user authentication by mouse-movement behavior in a continuous process and used an intensive dataset comprising 40 users for their evaluation. The study did a comparison between three machine learning models (SVM, KNN, Random Forest) and three Deep Learning models (1D-CNN, LSTM-RNN, ANN) and showed the considerable superiority of deep learning over traditional methods in terms of accuracy. The 1D-CNN was able to give an average accuracy of about 85%, while the ANN model went up to a maximum multi-class accuracy of 92.48%. This is a clear indication of the high potential of deep learning for behavioral biometric authentication through mouse dynamics. Thomas and Mathew (2022) proposed a Cat Recurrent Neural Model (CRNM) which integrates mouse dynamics and keystroke timing features for continuous authentication. The model successfully identifies intruders and achieved excellent results, such as 98.25% accuracy and a FAR of 0.01%, surpassing other methods in performance.

Wang et al. (2022) put forward SIURUA, a method for feature-fusion which fuses keystroke and mouse dynamics which leads to a hybrid-scene authentication accuracy of 84%. Shi et al. (2023) presented a method known as the Authentication Adaptation Network (AAN), which employs a heterogeneous domain adaptation approach to integrate keystroke and mouse dynamics through their projection into a shared subspace for subsequent classification as one. The technique, when applied to the real-life data of 41 users working on four different tasks, managed to achieve an accuracy of 89.22%, which is higher than that of traditional fusion models, such as UAMKL and Ensemble methods. Nevertheless, the technique requires extremely high feature dimensions, relies on lengthy 5-minute windows for optimal performance, and has been evaluated on a minor group of users under constrained conditions. Chandranegara et al. (2023) proposed a mouse-dynamics authentication technique that employed K-Nearest Neighbor (KNN) with simple random sampling, in order to improve user-attacker classification. The model reached an accuracy of 92% with a false acceptance rate (FAR) of 0.038 and a false rejection rate (FRR) of 0.068 when tested on the Balabit dataset, which was superior to the performance of CNN and decision-level fusion techniques. However, besides a relatively high FRR, the method is also based on simplified averaged features that might miss temporal patterns, and is established on just one dataset, which makes it less applicable in wider contexts.

In their paper, Roy et al. (2023) presented a new approach to mouse-dynamics authentication systems vulnerability testing where the fake user mouse-movement sequences are created with a novel Wasserstein Conditional DCGAN (WC-DCGAN). The model is capable of generating extremely realistic fake paths, which can deceive classifiers with an attack success rate of 69% in imitation attacks and even higher in surrogate attacks. Additionally, the research suggests a modified authentication strategy (MMDA) that will minimize misclassification errors and enhance robustness. Durmaz and Keskinöz (2023) improved the continuous mouse-dynamics authentication system by incorporating a ResNet-50 CNN, speeding up the mapping of images with the help of the features, and doing very realistic augmentation. It was shown that the method could reach 0.45 FAR and 0.34 FRR on the Balabit dataset, which means that it improved 6.5 times the past works. But the research is based on a small dataset of 10 users, utilizes the resource-demanding training of CNN, and has a separate model for each user, which could limit its scalability. Zhang and Bai (2024) put forward WiMouse, an authentication system that combines mouse movements with WiFi channel state information (CSI) by using PCA, Butterworth filtering, ReliefF, and SVM. Its performance was impressive, reaching 97.33% authentication and 93.90% identification accuracy in various indoor scenarios. Nevertheless, the system demands specialized WiFi equipment (IWL5300 NIC + router) and constant device positioning, thus restricting its usability.

AbouRida et al. (2024) found that LSTM achieved the highest accuracy among all the models used for continuous authentication, with 88% on keystrokes and 54% on mouse data, whereas XGBoost had a faster decision time. Szilágyi et al. (2024) suggested a mouse-dynamics authentication model based on LSTM, utilizing 23 detailed movement features from the DFL dataset. The model reached approximately 96% F1-score and approximately 99% AUC using 10 successive mouse movements, thus presenting very high performance similar to 20-movement models but with less overfitting. Nevertheless, the system requires numerous movements to come to a conclusion. The system for mouse dynamics authentication based on recurrence plots and the modified Vision Transformer was proposed by Mazumdar and Sundaram (2025) the model utilizing asymmetric recurrence plots and efficient attention managed to achieve AUC of 0.9812 and EER of 0.0498 on the Balabit dataset, 0.9919 AUC / 0.0220 EER on the DFL dataset, and 0.9791 AUC / 0.0546 EER on the Shen dataset. Thus, it outperformed the existing CNN, LSTM, and hybrid approaches.

Andrade et al. (2025) presented a DeepConvLSTM-based continuous authentication model that heavily relied on OS performance counters and recorded 99.73% correct identification, 0.28% false acceptance rate, and quick impostor detection of 10–17 s on real datasets. But system-level performance data dependence might trigger privacy issues. Xiong and Belman (2025) suggested a multi-modal adversarial activity detection method that combines keystroke dynamics and mouse-movement features through deep learning models (MLP, CNN, LSTM) and synchronized windows. The multi-modal LSTM reached 99% accuracy, which surpassed all uni-modal models and showed great potential in adversarial behavior detection, in an experiment involving 100 users with a total of more than 330,000 samples. However, the system faces the issue of massive data preprocessing and strict temporal alignment, which are the reasons that it might not be suitable for real-time deployment. Sa¨ıd et al. (2025a) proposed a frequency-domain approach using FDCT, Mellin transform, and Schur decomposition for robust feature embedding and secure data representation in medical images . Their work emphasizes extracting stable low-frequency features and enhancing robustness against transformations and noise. Jin and Liao (2025) developed a system based on mouse trajectory for performing both CAPTCHA farm detection and user authentication by finding out the similarity between two mouse movements employing a joint embedding network of (1D-CNN + LSTM). The model, which was trained on data from 130 users, got 96.9% AUC for CAPTCHA-farm (identity inconsistency) detection and 99.1% AUC for authentication, thus surpassing the best time-series models and mouse-based authentication systems, besides using only one classifier instead of per-user models. The system needs around 18.5 seconds of effective mouse movement to create a sample, which results in relatively high authentication latency in real-world scenarios. Beggari et al. (2025) proposed hybrid signal-processing frameworks using transform-domain techniques and clustering for robust feature representation. In another work (Sa¨ıd et al., 2025b), the authors employed Ridgelet transform and QR decomposition to extract directional and discriminative features from complex data, emphasizing stability and robustness.

Thomas and Keerikkattil (2025) employed feature-ranking techniques on keystroke and mouse-dynamic features and utilized a CRNM model for continuous authentication. The selection of the top-ranked features cut down the authentication time from 198 s to 138 s and increased the accuracy from 98.25% to 99.21%, thus the rare behavioral traits gave a stronger discrimination. On the other hand, the method is still dependent on long authentication periods and controlled datasets, which may restrict its usability and scalability in real-world applications. Aljoubory and Mahdi (2025) proposed a lightweight EfficientNet-inspired mouse-dynamics authentication model using engineered behavioral features and CGAN-generated synthetic users. The model achieved 99.24% accuracy, a macro F1-score of 0.991, and an inference time of 0.2331 s per sample on the SapiMouse dataset. However, the CGAN-generated synthetic impostor data may not fully represent sophisticated real-world mimicry attacks, and the use of fixed-size segments may limit the capture of longer-term behavioral patterns.

Federated Learning (FL) enables distributed model training while keeping data localized, thereby enhancing privacy in sensitive applications. However, it introduces several challenges, including communication overhead, system heterogeneity, statistical heterogeneity, and potential privacy leakage from shared model updates. Existing approaches such as Federated Averaging (FedAvg), local updating strategies, and model compression techniques have been proposed to improve communication efficiency and scalability (Li et al., 2020; Xie et al., 2024). Additionally, privacy-preserving mechanisms such as differential privacy and secure aggregation are widely used to protect user data during training (Guo et al., 2024; Karimireddy et al., 2020). To further address scalability and robustness issues in secure aggregation, Turbo-Aggregate has been proposed, which reduces aggregation complexity from quadratic to near-linear while ensuring privacy and robustness against user dropouts (So et al., 2021). Overall, these advancements highlight the importance of efficient and secure aggregation techniques in federated learning systems. Research on mouse dynamics has demonstrated considerable advancements, however, they still encounter major limitations like extending the period of authentication, inadequate defense against spoofing, restricted scalability, and privacy-preserving learning being non-existent. Our approach aims at bridging these gaps by employing Hybrid Transformer-CNN for reliable user identification, GAN for spoofing detection, Federated Learning for privacy, and FGSM-based adversarial robustness evaluation, which ultimately leads to a more effective, secure, and scalable authentication system.

### 2.1 Structure of the paper

The rest of the paper is structured in this way. The proposed Hybrid Transformer-CNN architecture is described in Section 3 along with feature engineering, preprocessing, behavioral analysis, and model parts. Section 4 contains the experimental analysis that comprises performance evaluation, computational complexity, ablation study, adversarial robustness, and federated learning with differential privacy. Finally, Section 5 concludes the paper with key findings and future research directions.

## 3 Hybrid transformer-CNN model architecture

The proposed Hybrid Transformer-CNN model enables real-time user authentication from engineered mouse-dynamics data. As shown in Figure 1, the workflow includes four stages: (1) feature engineering and preprocessing; (2) reshaping the feature vector with a channel dimension; (3) global behavior extraction using a Transformer encoder; and (4) local pattern learning using 1D CNN and pooling layers, followed by dropout and classification. This combined method effectively identifies long-range as well as short-range behavioral patterns making the authentication process accurate and quick. The proposed Hybrid Transformer-CNN model is designed to capture both global and local patterns in mouse dynamics data. The Transformer encoder consists of 2 layers with adaptive attention heads (up to 8) and a feedforward dimension of 128, ensuring a balance between model complexity and generalization. The CNN module includes two 1D convolutional layers with 32 and 64 filters (kernel size 3), followed by max-pooling, to capture local feature patterns. A dropout rate of 0.3 is applied to reduce overfitting. The model is trained using the Adam optimizer (learning rate = 0.001, weight decay = 1 × 10^−4^) with categorical cross-entropy loss for 120 epochs. Stratified 5-fold cross-validation is used for robust evaluation. SMOTE is applied to handle class imbalance, and Z-score normalization is used to standardize input features.

**Figure 1:**
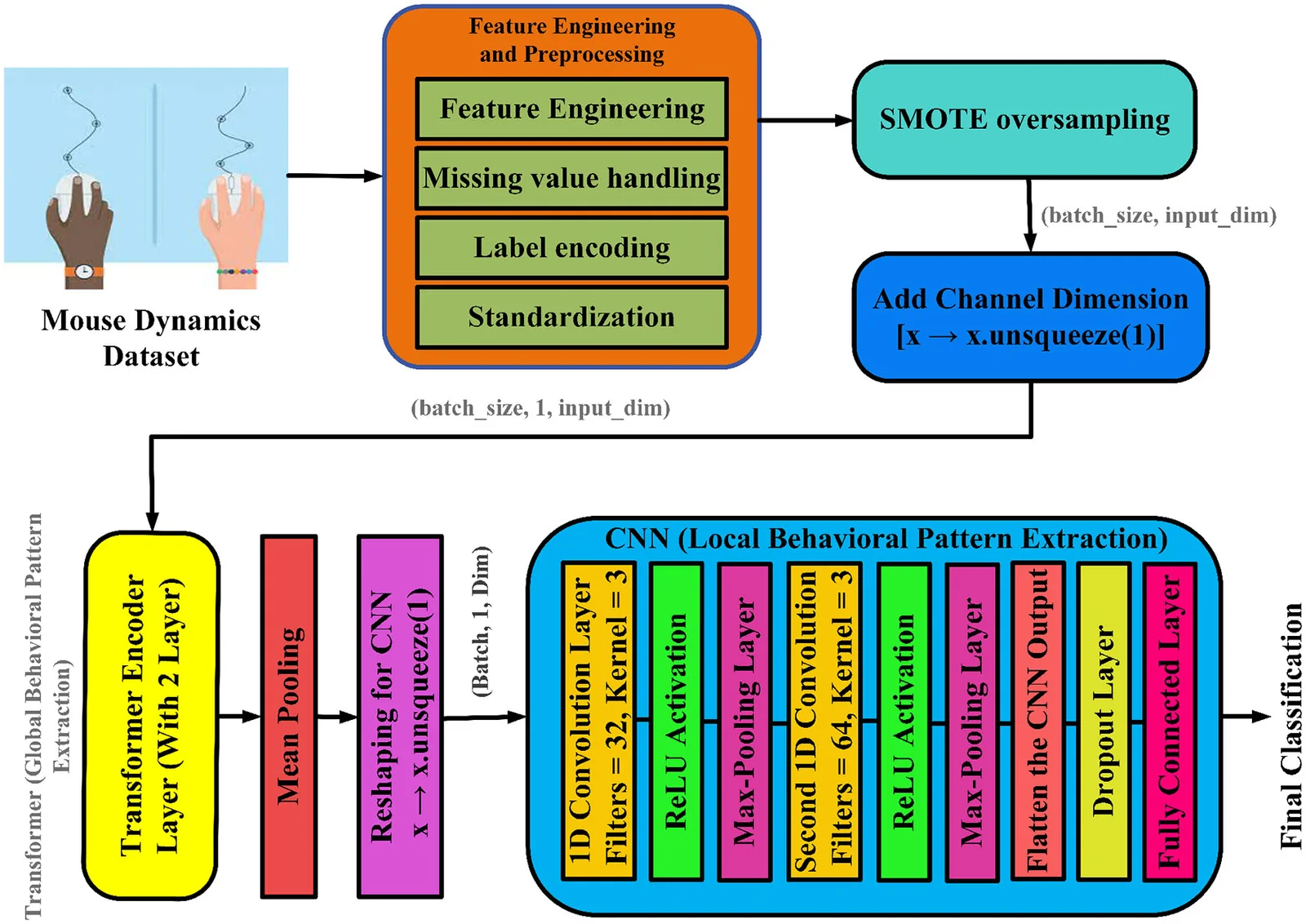
Architecture of the proposed Hybrid Transformer-CNN model.

### 3.1 Feature engineering

The original mouse-dynamics dataset contained only six basic fields: user ID, session ID, timestamp, screen position (x,y), and event type. These raw values do not reveal how a person actually uses the mouse. To convert this simple data into meaningful behavior patterns, a full feature-engineering process was applied. This process produced 14 new behavioral features, including timing features like time difference and idle time; movement features such as speed, acceleration, and jerk; path-based features like slope, path length, straight-line distance, and straightness; directional features such as angle change and direction-change count; interaction behavior through click density; and a PCA-based narrowness measure that shows how compact the mouse movements (Khan et al., 2024). These 14 engineered features (shown in Table 1) provide a much richer understanding of each user's mouse behavior and greatly improve the model's ability to distinguish users accurately. To further validate the independence of the engineered features, a correlation heatmap is generated (Figure 2). The results show that most features exhibit low to moderate correlation, indicating minimal redundancy. Higher correlations between certain feature pairs, such as time_diff and idle_time, and acceleration and jerk, are expected due to their inherent relationships.

**Table 1:** Engineered mouse-dynamics features.

Feature name	Feature definition	Mathematical formula
Time difference (Δ*t*)	Time gap between two consecutive mouse events.	Δ*t*_*i*_ = *timestamp*_*i*_−*timestamp*_*i*−1_
Speed (*v*)	Movement speed of pointer between two points.	$v_{i}=\frac{\sqrt{{\left(x_{i}-x_{i-1}\right)}^{2}+{\left(y_{i}-y_{i-1}\right)}^{2}}}{\text{Δ}t_{i}+1}$
Acceleration (*a*)	Change in cursor speed.	*a*_*i*_ = *v*_*i*_−*v*_*i*−1_
Jerk (*j*)	Change in acceleration between two events.	*j*_*i*_ = *a*_*i*_−*a*_*i*−1_
Slope (direction)	Direction of cursor movement using slope.	$m_{i}=\frac{y_{i}-y_{i-1}}{x_{i}-x_{i-1}+10^{-6}}$
Path length (PL)	Total cumulative distance traveled in a session.	$PL=\overset{n}{\underset{i=1}{\sum }}\sqrt{{\left(x_{i}-x_{i-1}\right)}^{2}+{\left(y_{i}-y_{i-1}\right)}^{2}}$
Straight-line distance (*D*_*straight*_)	Direct distance between start and end points of a session.	$D_{straight}=\sqrt{{\left(x_{end}-x_{start}\right)}^{2}+{\left(y_{end}-y_{start}\right)}^{2}}$
Straightness ratio (SR)	Efficiency of mouse movement (straightness).	$SR=\frac{D_{straight}}{PL+10^{-6}}$
Angle of movement (θ)	Angle of cursor movement between two points.	$\theta_{i}=\tan^{-1}\left(\frac{y_{i}-y_{i-1}}{x_{i}-x_{i-1}}\right)$
Angle change (Δθ)	Change in movement angle.	Δθ_*i*_ = θ_*i*_−θ_*i*−1_
Direction change count (DCC)	Number of sharp direction changes (>45°).	$DCC=\sum_{i=1}^{n}\left(|\text{Δ}\theta_{i}|>\frac{\pi }{4}\right)$
Idle time	Periods where mouse remains idle for more than 1 second.	$idle_{i}=\{\begin{array}{ll} \text{Δ}t_{i}, & \text{Δ}t_{i}>1\\ 0, & \text{otherwise} \end{array}$
Click density	Click frequency relative to total session events.	$click\text{\_}density=\frac{\text{click events}}{\text{total events}}$
Narrowness (PCA feature)	Compactness of cursor movement using PCA projection.	*N* = *PC*_1_(*screen*_*x, screen*_*y*)

**Figure 2:**
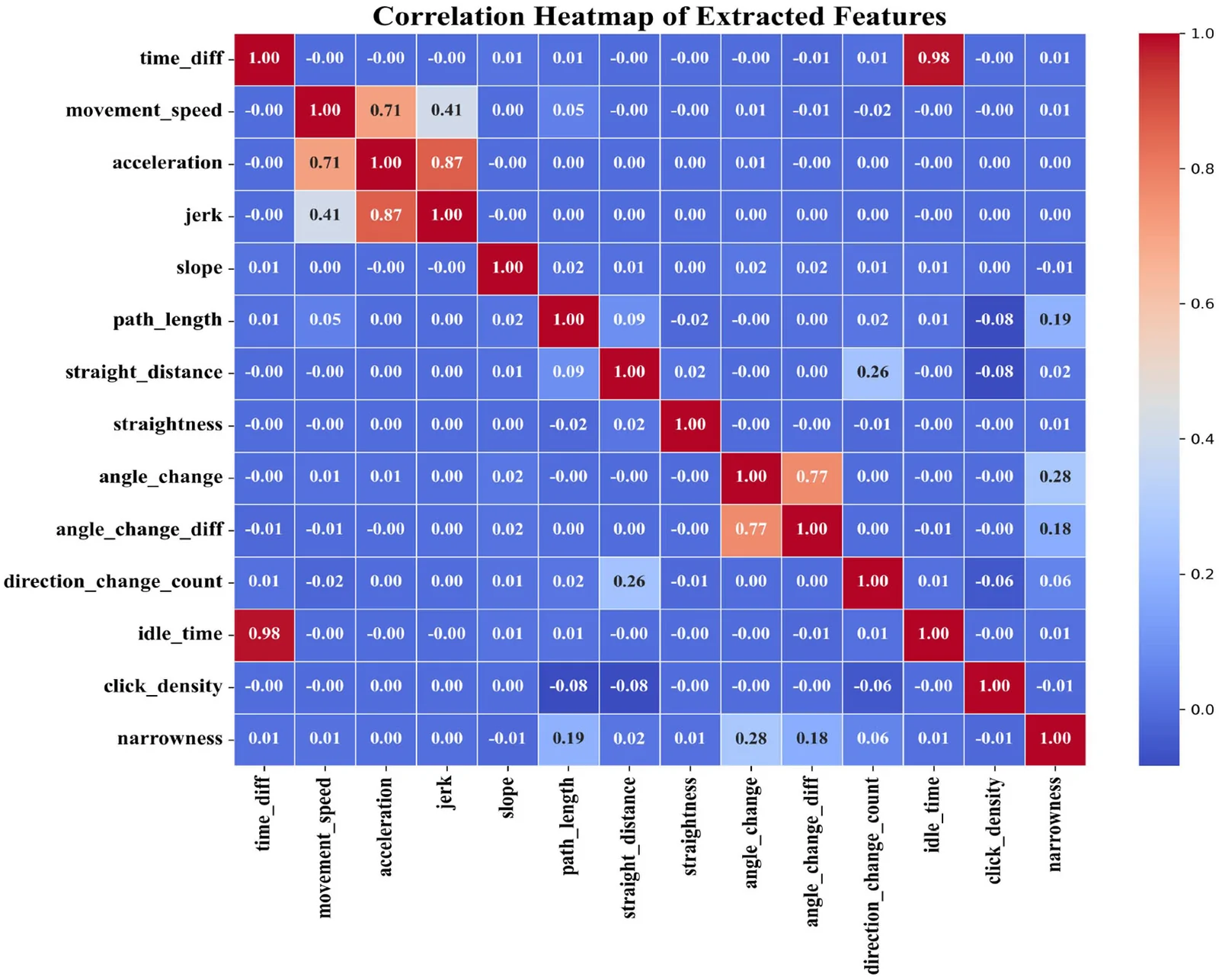
Correlation heatmap of the extracted mouse dynamics features.

### 3.2 Preprocessing

Mouse-dynamics data underwent a series of preprocessing steps to clean, transform, and get ready for model training. The data quality, consistency, and suitability for effective feature learning were ensured by these steps, with each component of the pipeline described in detail below.

1. **Remove identifier columns:** uid, session_id, and user_id were dropped in order to prevent data leakage and to make sure that only behavioral patterns are learned by the model.
2. **Handle missing values:** The missing entries were filled using median imputation, which is robust against outliers and therefore does not skew the feature distributions.
3. **Encode categorical features & labels:** Label encoding was applied, which transformed categorical attribute columns and the target variable (user_id) into numerical labels appropriate for deep learning models.
4. **Balance classes using SMOTE:** SMOTE was used to address class imbalance, increasing the training data from 11,633 to 15,400 samples and ensuring balanced user representation (Figure 3c). To prevent data leakage, SMOTE was applied only to the training data within each fold after data partitioning. Therefore, no synthetic samples generated from validation or test data were included during model training.

**Figure 3:**
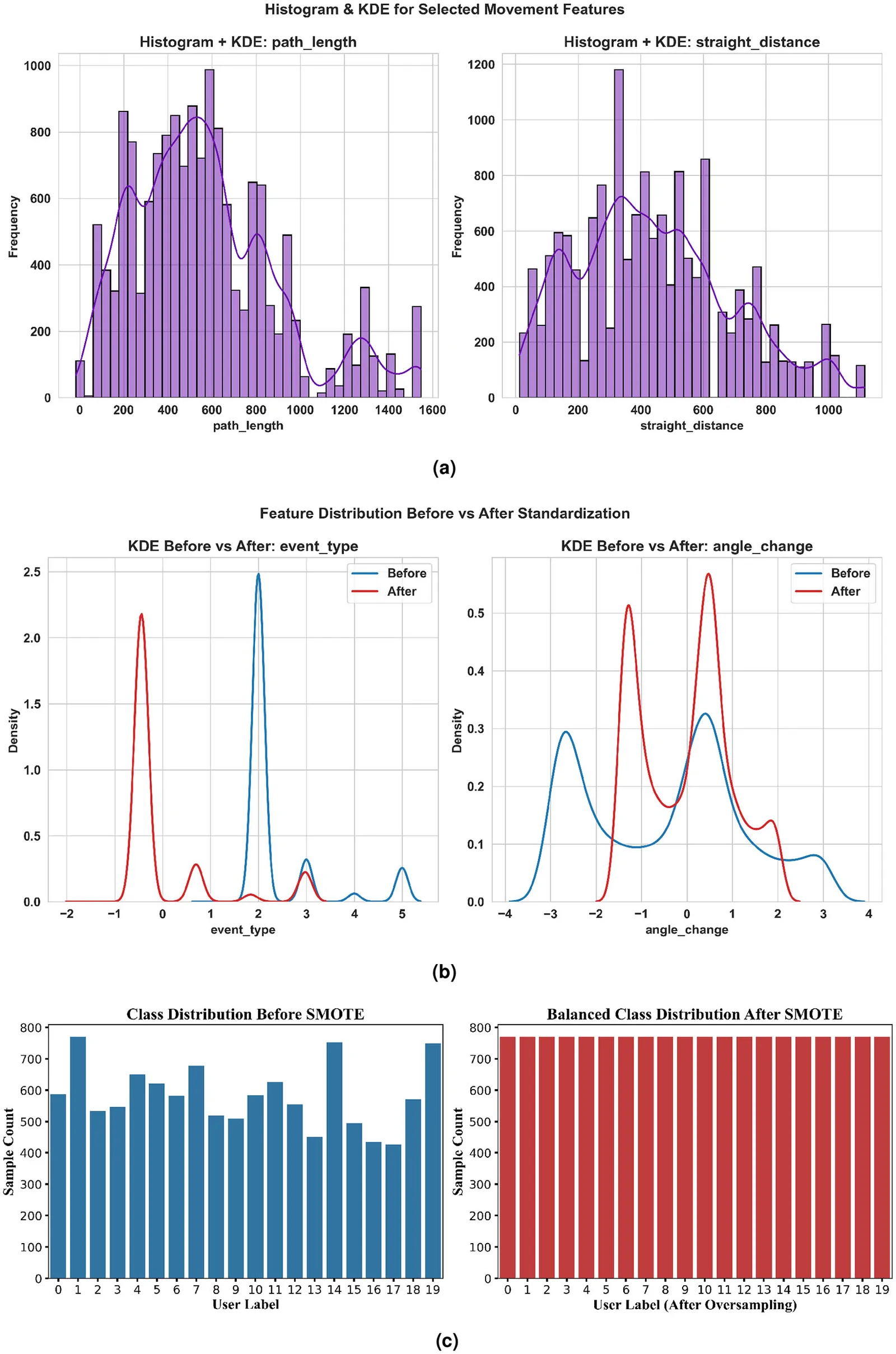
Preprocessing results showing **(a)** feature distributions, **(b)** normalization effects, and **(c)** balanced classes after SMOTE.

### 3.3 Behavioral analysis

1. **Leakage-controlled session-level stratified split:** stratified five-fold group cross-validation was applied, with approximately 80% of the data used for training and 20% for testing in each fold. A composite identifier combining the user ID and session ID was used to ensure that all samples and sequence windows belonging to one session were assigned exclusively to the training, validation, or test partition. No session or sequence window was shared across these partitions. The primary experiment followed a closed-set authentication protocol. Therefore, the same enrolled users were represented in both training and testing, while the test set contained only previously unseen sessions from those users. Mouse events were arranged chronologically according to their timestamps within each session, and fixed-length sequences were generated independently without crossing session boundaries. Missing-value imputation, feature encoding, standardization, and SMOTE were performed using only the training data. This procedure minimized session-specific memorization and provided a realistic evaluation of cross-session authentication performance.
2. **Standardization (Z-score):** applied Z-score normalization $z=\frac{x-\mu }{\sigma }$ to scale all features to zero mean and unit variance for stable model training (Figures 3a, b).
3. **Convert to PyTorch tensors:** all the features and labels that were preprocessed were converted into PyTorch tensors which made the training of the hybrid Transformer-CNN-Attention model on GPU more efficient.

The behavioral signals derived from mouse dynamics have strong statistical variations among different users and those differences are the basis for the proposed authentication framework. The combined visualizations–mouse trajectory, speed-acceleration-jerk profiles, angle-change dynamics, and cursor heatmaps–individually very well present the interaction patterns are showin in Figure 4. By mouse, users make different shapes, the smoothness of movement and cursor navigation tendencies are also different, which represent their personal motor-control habits. On the other hand, speed peaks, acceleration bursts, and jerk fluctuations vary as at different times the user will have their own rhythm of movement. Angle-change plots further show user-specific turning behavior, from smooth directional transitions to sharp corrective movements. Heatmap distributions also differ significantly, revealing that each user interacts with particular regions of the interface more frequently. The sum of these statistical deviations is the confirmation of capturing behavioral uniqueness in the extracted features and validation of their suitability as biometric indicators for continuous and robust user authentication.

**Figure 4:**
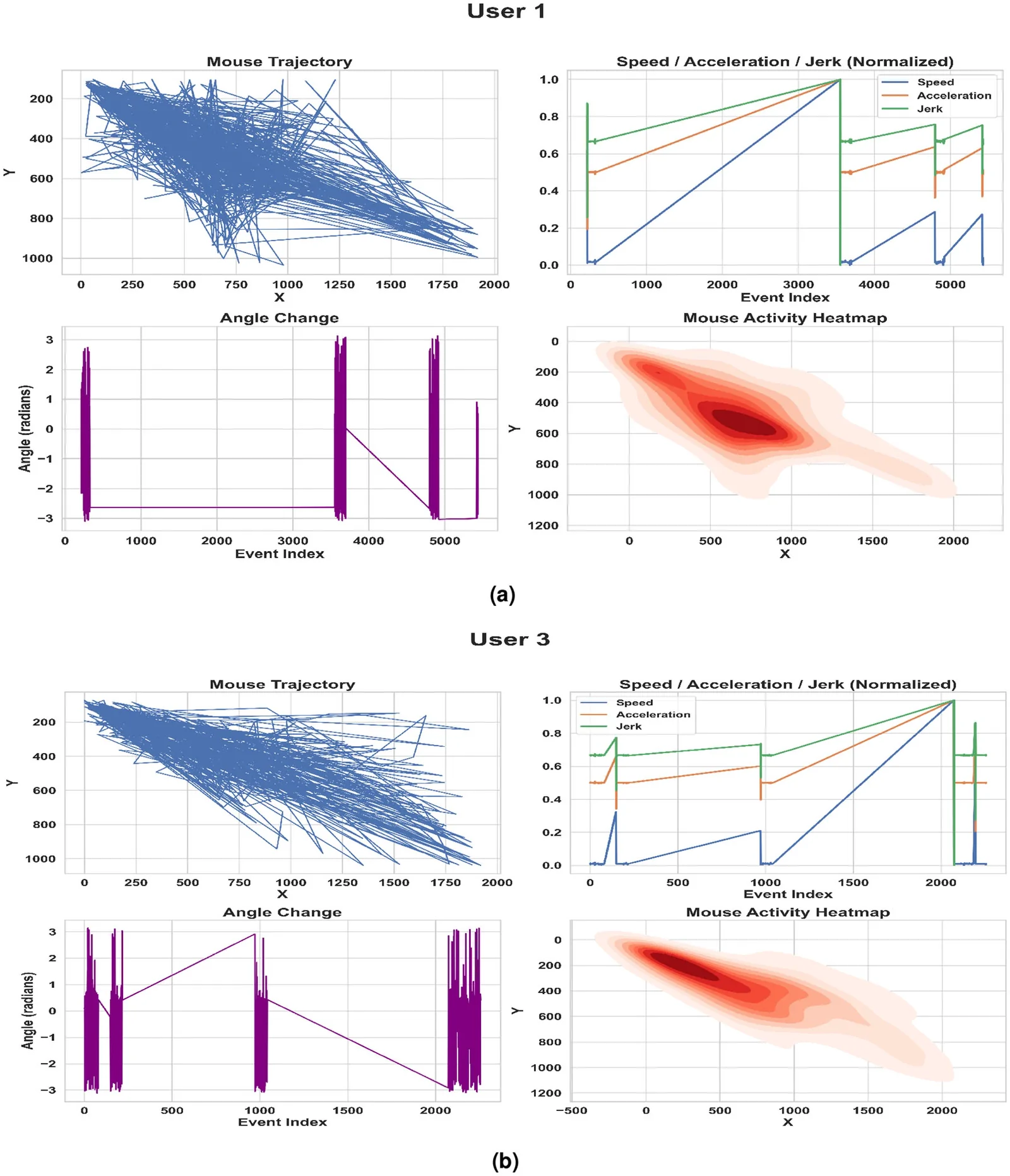
Mouse-dynamics behavioral patterns for **(a)** User 1 and **(b)** User 3. Each user is represented by four visualizations: mouse trajectory, normalized speed/acceleration/jerk, angle change, and mouse activity heatmap, illustrating user-specific mouse interaction patterns.

### 3.4 Input and data representation

After applying the SMOTE algorithm (Chawla et al., 2002), all the produced samples can be represented as vectors of fixed length D with dimension (batch, D). Next, a channel dimension is added (unsqueeze) to the already existing vector to get it shaped (batch, 1, D) which is suitable for the Transformer. The Transformer then outputs the tensor in shape (batch, D) which is squeezed again and reshaped to (batch, 1, D) before passing through the 1D CNN layers.

### 3.5 Transformer encoder

The Transformer encoder block is used to capture global behavioral dependencies throughout the engineered feature space. The input to this block is structured as (batch, 1, D), treating each feature dimension as a separate token. The encoder is made up of two stacked Transformer layers, as shown in Figure 5. Each has multi-head self-attention, a position-wise feedforward network (with dim_feedforward = 128), layer normalization, and dropout (0.3). The number of attention heads is deterministically chosen using max[1, min(8, D/)8] to adjust to the feature dimensionality. The encoder keeps the spatial shape (batch, 1, D), and afterwards the output is squeezed to (batch, D) to give a compact representation for the next 1D CNN module. To further enhance the modeling capability, a sequence-based formulation is also considered to capture temporal behavioral patterns. In this approach, consecutive mouse events are grouped using a sliding window mechanism, forming input samples of shape (batch, sequence length, feature dimension). This enables the Transformer to learn dependencies across time-ordered events in addition to feature-level relationships.

**Figure 5:**
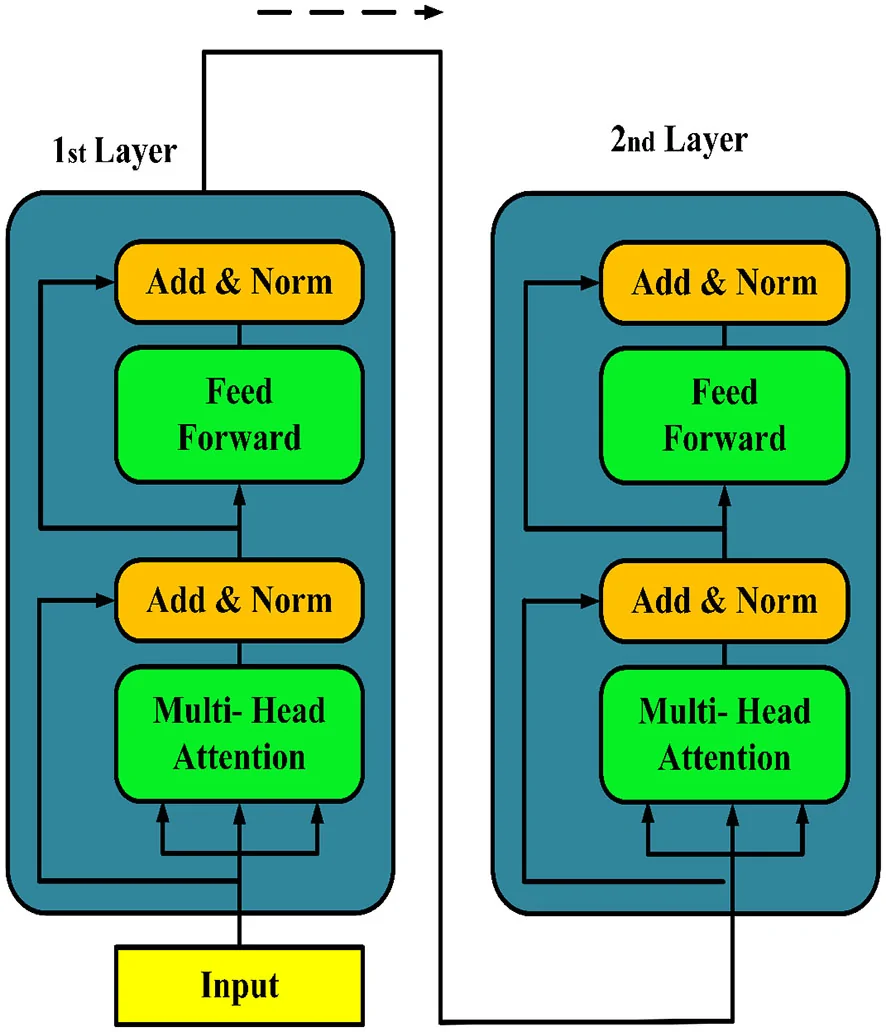
Transformer encoder with two stacked layers.

### 3.6 1D CNN block and classification head

The 1D CNN block is responsible for identifying the local behavioral micro-patterns that include short acceleration bursts and subtle changes in curvature. The block is composed of two convolution layers, where the first one has 32 filters and the second one has 64 filters, and both layers use a kernel size of 3 with padding 1 and are followed by ReLU activation and MaxPool1d (kernel size 2, stride 2). The feature maps that come out of these operations are then flattened to get a compact representation. A dropout layer (p = 0.3) is used to mitigate overfitting and then a fully connected layer transforms the features that have been flattened to the number of user classes. During the inference phase, softmax is applied to get the class probabilities. The model operation is based on CrossEntropyLoss and the Adam optimizer (learning rate 1e-3, weight decay 1e-4) and trained for 120 epochs per fold.

The overall workflow of the mouse dynamics authentication framework proposed is illustrated in Figure 6, where the different stages from feature engineering and preprocessing to Hybrid Transformer-CNN-based classification and authentication decision are shown in sequence.

**Figure 6:**
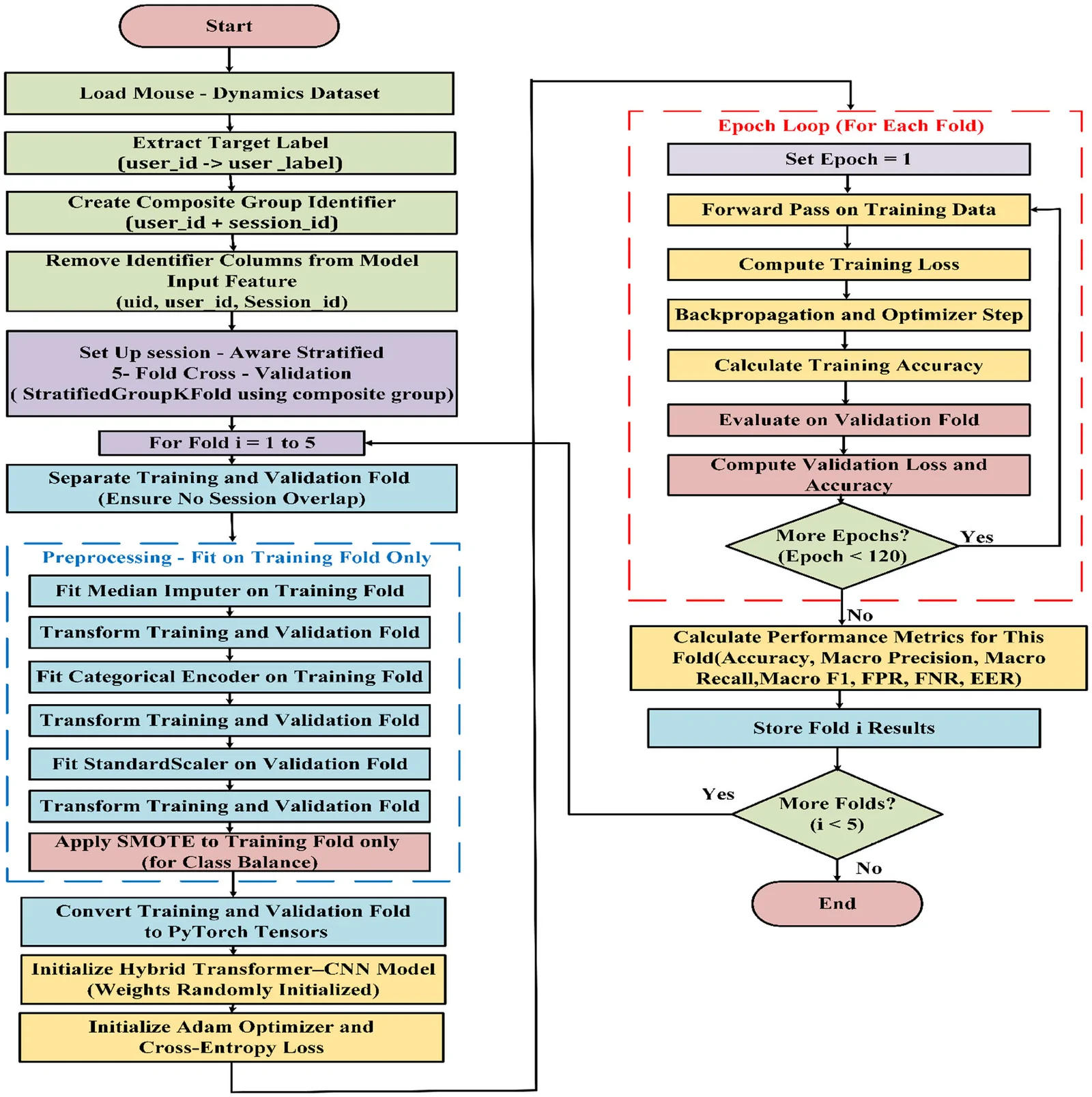
Proposed model flow chart.

### 3.7 Implementation details and hyperparameter configuration

The input to the proposed model is represented as a tensor of size *B*×20 × 21, where *B* denotes the batch size, 20 is the sequence length, and 21 is the number of mouse-dynamics features. Each 21-dimensional feature vector is projected into a 128-dimensional embedding using a linear projection layer. A learned positional embedding of size 1 × 20 × 128 is added to the projected features to preserve the order of mouse events. The Transformer module consists of two encoder layers with eight self-attention heads. The model dimension is 128, resulting in 16 dimensions per attention head, while the feed-forward dimension is 128. Each Transformer encoder layer uses ReLU activation, a dropout rate of 0.30, and post-layer normalization with a LayerNorm epsilon of 10^−5^. The Transformer output is transposed and passed through two one-dimensional convolutional layers with 32 and 64 output channels, respectively. Both convolutional layers use a kernel size of 3, stride of 1, padding of 1, and ReLU activation. Each convolution is followed by max pooling. Adaptive average pooling reduces the extracted representation to 64 features, which are passed through dropout and a fully connected classification layer with 20 outputs. The complete model contains 224,372 parameters, all of which are trainable. Table 2 summarizes the architecture and training hyperparameters.

**Table 2:** Architecture and hyperparameter configuration of the proposed Hybrid Transformer–CNN model.

Component/parameter	Configuration	Output dimension
Input sequence	21 mouse-dynamics features; sequence length = 20	*B*×20 × 21
Input projection	Linear transformation: 21 → 128	*B*×20 × 128
Positional encoding	Learned positional embedding of size 1 × 20 × 128; initialized using N(0,0.02)	*B*×20 × 128
Transformer encoder	2 encoder layers, 8 attention heads, *d*_model_ = 128, and 16 dimensions per head	*B*×20 × 128
Feed-forward network	Dimension = 128; ReLU activation	*B*×20 × 128
Normalization	Post-LayerNorm; LayerNorm epsilon = 10^−5^	*B*×20 × 128
Transformer dropout	Dropout rate = 0.30	*B*×20 × 128
Tensor transpose	Transformer output rearranged for one-dimensional convolution	*B*×128 × 20
Conv1D layer 1	128 → 32 channels; kernel = 3; stride = 1; padding = 1; ReLU	*B*×32 × 20
Max-pooling layer 1	Kernel size = 2; stride = 2	*B*×32 × 10
Conv1D layer 2	32 → 64 channels; kernel = 3; stride = 1; padding = 1; ReLU	*B*×64 × 10
Max-pooling layer 2	Kernel size = 2; stride = 2	*B*×64 × 5
Adaptive average pooling	Output size = 1	*B*×64 × 1
Flattening and dropout	Flattening followed by dropout of 0.30	*B*×64
Classification layer	Fully connected layer with 20 user classes	*B*×20
Trainable parameters	224,372	–
Sequence stride	1	–
Optimizer	Adam	–
Learning rate	1 × 10^−3^	–
Weight decay	1 × 10^−4^	–
Loss function	Cross-entropy loss	–
Batch size	128	–
Number of epochs	120	–
Evaluation protocol	Session-disjoint stratified five-fold cross-validation	–
SMOTE	Applied only to training sequences within each fold	–
Random seed	42	–

## 4 Experimental analysis

### 4.1 Experimental setup and dataset details

The experiments were performed using Jupyter Notebook on a local computer with an 11th Gen Intel Core i7-1165G7, 16 GB RAM, and a 64-bit Windows operating system. Python 3.12 was utilized along with the main libraries of PyTorch, scikit-learn, Pandas, NumPy, and Matplotlib while psutil was used to track memory and runtime performance. The proposed Hybrid Transformer-CNN model was trained and evaluated on a mouse-dynamics dataset (Jaafar, 2021) that included multi-session cursor-movement records from several users, which contained X-Y coordinates with time stamps, types of movements, and clicks as actions. The conversion of these raw events into high-level behavioral features was done to facilitate the user-authentication modeling process.

### 4.2 Evaluation metrics

In the present research, standard evaluation metrics were applied in the form of Accuracy, Precision, Recall, and F1-Score. Moreover, the models were further evaluated using the following measures: FPR, FNR, Training Time, Prediction Time and Equal Error Rate (EER) (Naidu et al., 2023).

1. **Accuracy:** Measures the overall proportion of correctly classified samples across all classes. $\text{Accuracy}=\frac{\sum_{i=1}^{C}TP_{i}}{\sum_{i=1}^{C}\left(TP_{i}+FP_{i}+FN_{i}\right)}$
2. **Precision (macro):** Indicates how many predicted positive samples for each class are actually correct, averaged across classes.

${\text{Precision}}_{macro}=\frac{1}{C}\sum_{i=1}^{C}\frac{TP_{i}}{TP_{i}+FP_{i}}$

3. **Recall (macro):** Measures the model's ability to correctly identify actual class samples, averaged across classes.

${\text{Recall}}_{macro}=\frac{1}{C}\sum_{i=1}^{C}\frac{TP_{i}}{TP_{i}+FN_{i}}$

**4. F1-Score (macro):** Harmonic mean of precision and recall, computed for each class and averaged.

${\text{F1}}_{macro}=\frac{1}{C}\sum_{i=1}^{C}\frac{2TP_{i}}{2TP_{i}+FP_{i}+FN_{i}}$

**5. False Positive Rate (FPR—macro):** Represents how often the model incorrectly predicts a class when it does not belong to that class (impostor acceptance).

${\text{FPR}}_{macro}=\frac{1}{C}\sum_{i=1}^{C}\frac{FP_{i}}{FP_{i}+TN_{i}}$

**6. False Negative Rate (FNR—macro):** Indicates the rate at which the model fails to detect genuine class samples (genuine user rejection).

${\text{FNR}}_{macro}=\frac{1}{C}\sum_{i=1}^{C}\frac{FN_{i}}{TP_{i}+FN_{i}}$

**7. Equal Error Rate (EER):** EER represents the position at which the FPR is equal to the FNR. In the case of multiclass problems, EER is determined by averaging the values obtained from the one-vs-rest ROC curves of each class.

EER = FPR whereFPR = 1 − TPR

### 4.3 Experimental details

The comparison of different baseline models and hybrid architectures on the authentication dataset is shown in Table 3. The traditional sequential models, like LSTM and GRU, got a moderate performance, with accuracies of 42.77% and 52.36% respectively. This was due to their limited ability to represent mouse-dynamics in a spatial-temporal pattern. Then, the accuracy of MLP and GNN models increased, e.g., 58.29% and 65.03% respectively.

**Table 3:** Performance comparison of baseline models and the proposed model.

Model	Accuracy	Precision	Recall	F1 Score	FPR	FNR	EER
LSTM	0.4277	0.4251	0.4277	0.3957	0.03012	0.57227	0.045455
GRU	0.5236	0.5251	0.5236	0.5096	0.025072	0.47636	0.023103
MLP	0.5829	0.5934	0.5829	0.5657	0.021951	0.41708	0.013260
GNN	0.6503	0.6580	0.6503	0.6455	0.018404	0.34968	0.014354
Transformer + GNN	0.3806	0.3941	0.3806	0.3294	0.032601	0.61942	0.011210
GRU + Transformer	0.7010	0.7159	0.7010	0.7011	0.015738	0.29903	0.408202
GRU + MLP	0.7631	0.7745	0.7631	0.7625	0.012471	0.23695	0.070586
MLP + GNN	0.8133	0.8213	0.8133	0.8115	0.009826	0.18669	0.051256
LSTM + CNN	0.8479	0.8576	0.8479	0.8492	0.008008	0.15214	0.004580
CNN + GNN	0.8484	0.8546	0.8484	0.8488	0.007980	0.15162	0.006015
**Proposed Model**	**0.9951**	**0.9953**	**0.9951**	**0.9951**	**0.000256**	**0.00487**	**0.000068**

Bold values indicate the best performance obtained among all compared models for the corresponding evaluation metric.

The evaluated hybrid models showed different levels of performance depending on the complementary characteristics of their components. GRU + MLP, MLP + GNN, and LSTM + CNN achieved accuracies of 76.31%, 81.33%, and 84.79%, respectively. These results indicate that hybridization can improve discriminative capability when the combined models capture complementary behavioral information, rather than simply because multiple techniques are integrated. The CNN + GNN model achieved a comparable accuracy of 84.84%, suggesting that graph-based feature propagation can also effectively represent relationships within mouse-dynamics features.

The proposed model demonstrates strong performance compared with the baseline methods, achieving an accuracy of 99.51%, precision of 99.53%, and recall of 99.51%, together with a low FPR of 0.000256 and an EER of 0.000068. These results indicate that the framework is effective in distinguishing legitimate users from impostor behavior. The low FNR of 0.00487 further demonstrates its ability to minimize false rejections, which is important for practical authentication systems. As shown in Figures 7a, b, the training and validation accuracies increase steadily, while the corresponding losses decrease consistently, indicating stable convergence without significant overfitting. The confusion matrix presented in Figure 8 shows only a small number of misclassifications, further confirming the strong discriminative capability of the proposed model.

**Figure 7:**
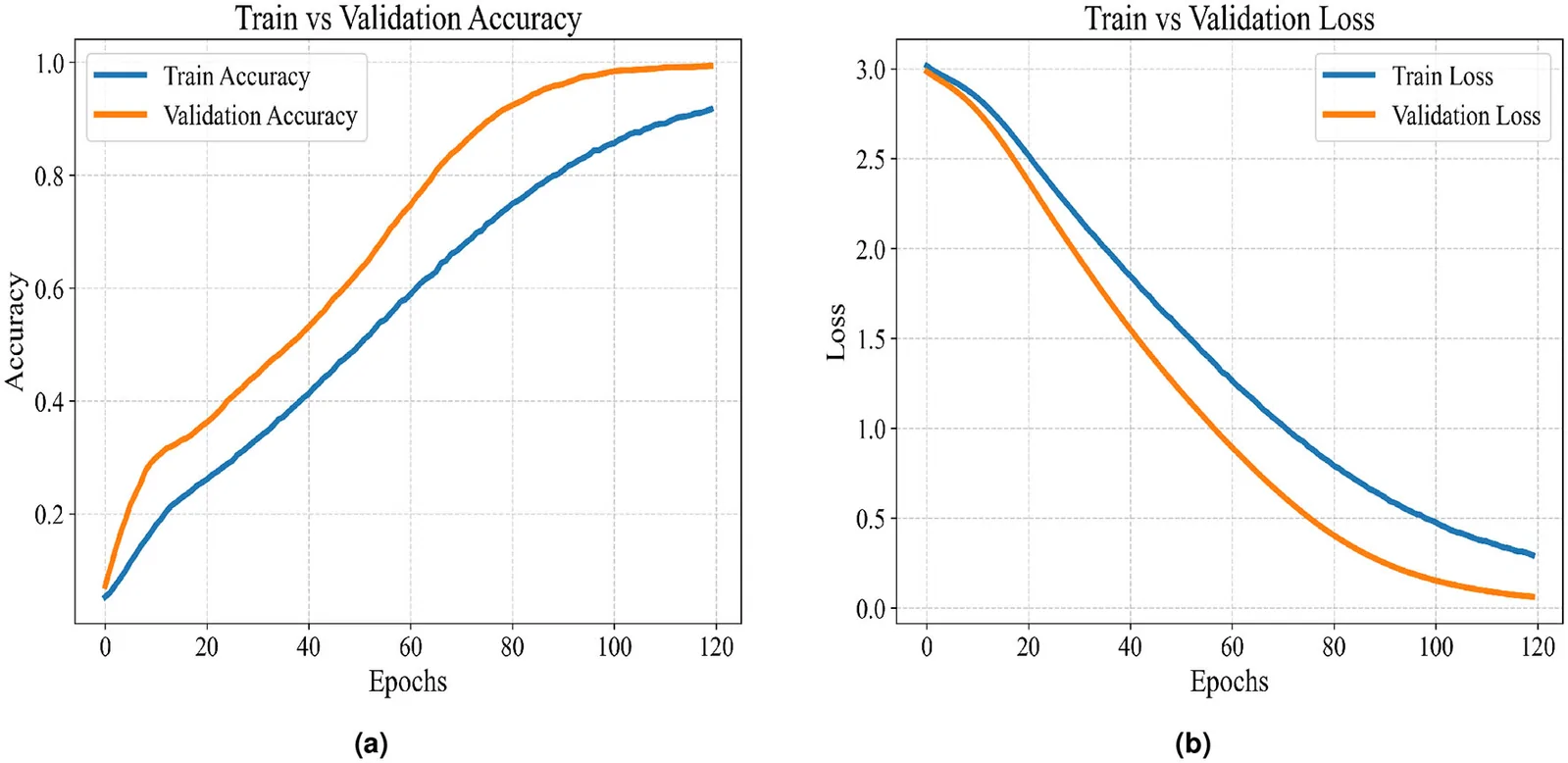
Model training performance curves: **(a)** Training vs. validation accuracy curve; **(b)** Training vs. validation loss curve.

**Figure 8:**
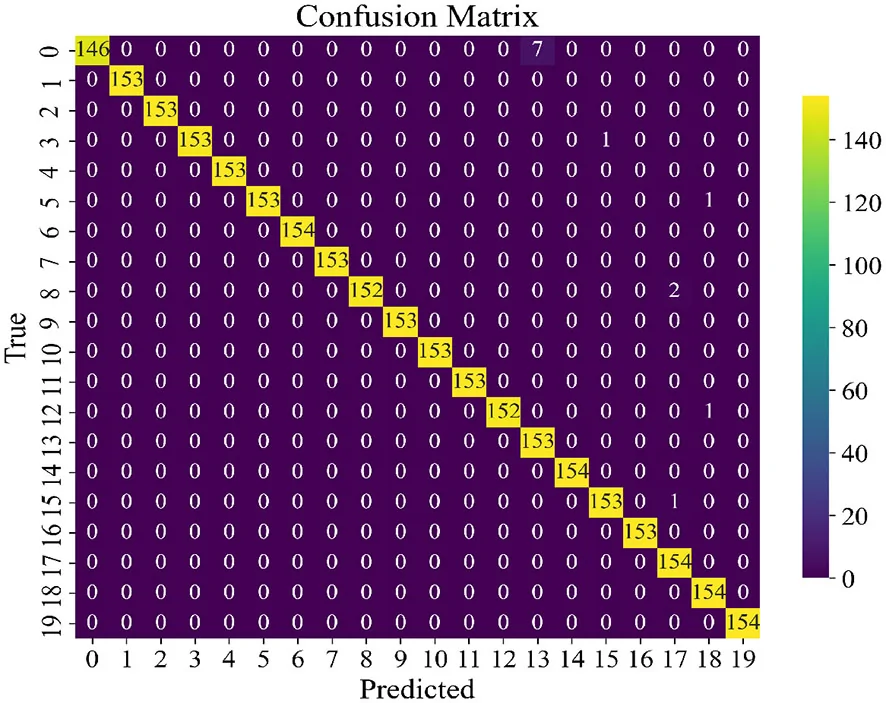
Confusion matrix of the proposed model.

These results were obtained under a closed-set, session-disjoint evaluation protocol. Therefore, the model identified enrolled users from previously unseen sessions rather than completely unseen users. The high performance can be attributed to the discriminative engineered features and the complementary capabilities of the Transformer and CNN components in learning long-range temporal dependencies and local movement patterns. Nevertheless, the reported results are specific to the evaluated dataset and experimental setting and should not be interpreted as universal performance across unseen devices, applications, environments, or user populations.

To incorporate temporal information, mouse events were grouped using a sliding-window mechanism, in which consecutive events within each session were aggregated into fixed-length sequences. No sequence was allowed to cross a session boundary. Each sample was therefore represented as a sequence of feature vectors, enabling the model to learn time-dependent behavioral patterns. To examine the influence of temporal context, experiments were conducted using different sequence lengths. A window size of 10 achieved an accuracy of 99.55%, while a window size of 20 produced the highest accuracy of 99.75%. Increasing the window size to 30 resulted in an accuracy of 99.69%. These findings indicate that a sequence length of 20 provided the most effective temporal context among the evaluated window sizes for capturing user-specific mouse behavior.

### 4.4 Computational efficiency analysis

The training time, inference time, detection latency per sample and reaction time were used as the metrics to evaluate the computational efficiency (Polson and Sokolov, 2020) of all baseline models and the proposed architecture. Table 4 shows that the proposed model gets a very low inference time and detection latency of 0.000037 seconds per sample making it appropriate for real-time authentication. The training time is longer because of the model complexity but the reaction time is still very low (0.037 ms) which proves that the proposed framework achieves high accuracy at the same time retaining runtime performance.

**Table 4:** Computational performance comparison of baseline models and the proposed model.

Model	Training time (Sec)	Inference time (Sec)	Detection time (Sec/Sample)	Reaction time (ms/Sample)	Peak memory (MB)	FLOPs (MFLOPs)
LSTM	22.307	0.019307	0.000006	0.006	466.03	0.21
GRU	13.811	0.013011	0.000004	0.004	538.75	0.16
MLP	1.889	0.001201	0	0	194.71	0.01
GNN	4.057	0.004731	0.000002	0.002	538.75	0.16
Transformer + GNN	11.487	0.030003	0.000010	0.010	740.82	0.28
GRU + Transformer	48.038	0.119172	0.000039	0.039	898.27	0.43
GRU + MLP	17.915	0.015641	0.000005	0.005	539.29	0.18
MLP + GNN	6.937	0.009881	0.000003	0.003	772.78	0.24
LSTM + CNN	65.029	0.000016	0.000016	0.016	1155.87	0.64
CNN + GNN	21.346	0.000012	0.000012	0.012	1209.85	0.79
**Proposed model**	**53.184**	**0.000037**	**0.000037**	**0.037**	**492.42**	**0.08**

Bold values indicate the computational performance results of the proposed model.

Although the proposed model combines Transformer and CNN modules, its computational cost remains low because of the compact two-layer Transformer, 128-dimensional representation, lightweight 1D convolutions, and pooling-based feature reduction. As a result, the model requires only 0.08 MFLOPs while achieving high authentication performance. The peak memory usage of 492.42 MB reflects a trade-off between memory consumption and predictive accuracy, but remains lower than several larger hybrid baselines. The low FLOPs and reaction time of 0.037 ms/sample indicate that the model is suitable for real-time deployment on edge platforms with moderate resources. For highly constrained embedded devices, further optimization through pruning, quantization, or model compression may be required.

### 4.5 Open-Set evaluation with per-user thresholding

The proposed model is first evaluated using a closed-set multi-class approach. However, in real-world continuous authentication systems, unknown users (impostors) may appear. To handle this, the evaluation is extended to an open-set authentication scenario. In this case, the model must identify genuine users and reject unknown users. The open-set performance of the model is shown in Table 5, which includes FAR, FRR, and EER values across different folds. These results provide a more practical and realistic evaluation of the system. A threshold sensitivity analysis is performed to identify the optimal operating points for authentication. Different threshold values are tested to observe their impact on False Acceptance Rate (FAR), False Rejection Rate (FRR), and Equal Error Rate (EER). The results are shown in Table 5, where at a threshold of 0.3, the FAR is 0.0001 and FRR is 0.0010, while at higher thresholds such as 0.9 and 0.95, the FAR reduces to 0.0000 and the FRR increases to 0.0017 and 0.0026, respectively. The EER remains consistently low at 0.0001 across all threshold values, indicating stable and reliable performance of the proposed model.

**Table 5:** Open-set performance and threshold sensitivity analysis of the proposed model.

Open-set performance			
Fold	FAR	FRR	EER
Fold-1	0.0028	0.0019	0.0028
Fold-2	0.0001	0.0010	0.0001
Fold-3	0.0000	0.0000	0.0000
Fold-4	0.0002	0.0003	0.0002
Fold-5	0.0002	0.0010	0.0002
Avg	0.00066	0.00084	0.00066
Threshold analysis			
Threshold	FAR	FRR	EER
0.3	0.0001	0.0010	0.0001
0.5	0.0000	0.0010	0.0001
0.7	0.0000	0.0011	0.0001
0.9	0.0000	0.0017	0.0001
0.95	0.0000	0.0026	0.0001

Per-user operating points are used to improve practical authentication, as each user has unique behavioral patterns. Instead of a single global threshold, individual thresholds are applied for better discrimination. As shown in Table 6, baseline models exhibit a significant increase in FRR under per-user settings. In contrast, the proposed Hybrid model achieves 0.9951 accuracy (FAR = 0.0000, FRR = 0.0008) in global mode and 0.9872 accuracy (FAR = 0.0037, FRR = 0.2046) in per-user mode. Despite a slight performance drop, the proposed model maintains a better balance between security and usability, demonstrating its effectiveness for real-world authentication.

**Table 6:** Comparison of global and per-user threshold performance across models.

Model	Type	Accuracy	FAR	FRR
LSTM + CNN	Global	0.8788	0.0015	0.2945
	Per-user	0.9575	0.0012	0.8421
CNN + GNN	Global	0.7671	0.0025	0.4856
	Per-user	0.9518	0.0012	0.9463
MLP + GNN	Global	0.5836	0.0028	0.6891
	Per-user	0.9538	0.0015	0.9068
Proposed Hybrid	Global	0.9951	0.0000	0.0008
	Per-user	0.9872	0.0037	0.2046

### 4.6 Ablation study on proposed transformer-CNN model

To evaluate the contribution of each architectural component, an ablation study was carried out on the proposed Transformer-CNN model (Table 7). The independent Transformer and CNN variants reached accuracies of 59.97% and 89.19% respectively, which implies that neither architecture separately is capable of yielding high-performance authentication. The combination of all configurations of Transformer+CNN resulted in a remarkable enhancement, as the accuracies exceeded 98% in all cases. The elimination of dropout or SMOTE brought about a performance decline that was very clear and this confirmed their roles in regularization and class-balance handling. The experiments with varying CNN kernel sizes (3, 5, and 7) indicated that the changes in kernel size do not affect the performance, with kernel sizes 3 and 7 giving the best accuracy. Likewise, the testing with varying counts of attention heads showed that when only one head is used, the inference time (0.000017 seconds) is the shortest, but accuracy is not affected. All in all, the results of the ablation study confirm that the combination of components is responsible for the excellent performance of the proposed hybrid model.

**Table 7:** Ablation study: accuracy and inference time.

Variant	Accuracy	Inference time (Sec)
Transformer only	0.5997	0.101465
CNN Only	0.8919	0.090855
No Dropout	0.9944	0.000035
No SMOTE	0.9882	0.000036
Kernel = 3	0.9951	0.000038
Kernel = 5	0.9915	0.000037
Kernel = 7	0.9944	0.000037
Heads = 1	0.9922	0.000017
Heads = 2	0.9948	0.000035
Layers = 1	0.9820	0.000038
Layers = 2 (Proposed)	0.9951	0.000035

### 4.7 Model robustness and statistical validation

To assess the stability and robustness (Rainio et al., 2024) of the proposed model, we executed multiple training runs and k-fold cross-validation (Table 8). The model reached a mean accuracy of 0.9941 with a very low standard deviation of 0.0003, indicating highly stable training behavior, across three independent runs. In addition, the 5-fold cross-validation produced a mean accuracy of 0.9935 (SD = 0.0019), whereas the 10-fold CV reached 0.9944 (SD = 0.0026). The tight 95% confidence intervals in both instances confirm that the model is capable of generalizing consistently among varying data splits. Furthermore, statistical significance testing is performed using a paired t-test between the proposed Hybrid model and baseline models (LSTM + CNN, CNN + GNN, and MLP + GNN), where all comparisons yield p-values less than 0.001, confirming that the observed performance improvements are statistically significant.

**Table 8:** Model stability and cross-validation performance.

Evaluation type	Accuracy
Repeated runs (3 runs)	0.9941 ± 0.0003
5-Fold CV mean accuracy	0.9935
5-Fold CV Std Dev	0.0019
5-Fold CV 95% CI	[0.9912, 0.9959]
10-Fold CV mean accuracy	0.9944
10-Fold CV Std Dev	0.0026
10-Fold CV 95% CI	[0.9926, 0.9963]

Paired statistical comparisons were conducted using accuracy values obtained from identical five-fold cross-validation partitions. The paired *t*-test assumes correctly matched observations, approximately normally distributed paired differences, and independence of those differences. Normality was assessed using the Shapiro–Wilk test and was not rejected for the CNN+GNN, LSTM+CNN, and MLP+GNN comparisons, with *p*-values of 0.3705, 0.2560, and 0.3185, respectively. Therefore, paired *t*-tests were applied. The proposed model achieved mean improvements of 14.78, 14.83, and 18.29 percentage points over CNN+GNN, LSTM+CNN, and MLP+GNN, respectively. The corresponding 95% confidence intervals were 14.22–15.33, 14.28–15.38, and 17.75–18.83 percentage points, none of which included zero. After Holm–Bonferroni correction for multiple comparisons, the adjusted *p*-values remained statistically significant at 3.80 × 10^−7^, 3.80 × 10^−7^, and 2.33 × 10^−7^, respectively. The corresponding Cohen's *d*_*z*_ values were 33.10, 33.52, and 41.91, indicating numerically very large effects. However, because cross-validation folds use overlapping training data and only five paired observations were available, complete independence cannot be guaranteed. Therefore, the statistical results and effect sizes should be interpreted cautiously.

### 4.8 Adversarial robustness and evaluation

Adversarial robustness in machine learning, particularly in deep learning models, refers to the ability of a system to maintain reliable performance when subjected to carefully crafted perturbations designed to mislead predictions (Liang et al., 2020). This property is crucial in safety-critical applications such as healthcare, autonomous systems, and cybersecurity. The proposed Hybrid Transformer-CNN model is evaluated under FGSM, PGD, and CW-L2 adversarial attacks, as summarized in Table 9. All attacks are performed in an untargeted setting, where the objective is to induce general misclassification. The perturbation levels (ϵ) are applied on Z-score normalized features, ensuring consistent scaling across inputs. The adversarial robustness evaluation was performed under an untargeted white-box setting, where the attacker had access to the trained model and its gradients. FGSM generated adversarial samples using a single gradient-sign update. PGD was implemented as an iterative *L*_∞_-bounded attack using 10 iterations, with adversarial samples projected back into the permitted perturbation range after each update. CW-L2 was implemented as an optimization-based *L*_2_-constrained attack using a fixed number of iterations. All attacks used fixed iteration settings without early stopping, and an attack was considered successful when a correctly classified clean sample was misclassified after perturbation. During adversarial training, adversarial samples were generated from the training data using the current model and incorporated into the training process to improve robustness. The computational overhead of adversarial training was quantified using the additional training time compared with standard training, while the inference procedure remained unchanged.

**Table 9:** Stress-testing analysis of the proposed model under adversarial attacks with adversarial training.

Attack Type	Attack parameters	Original accuracy (%)	Post-attack accuracy (%)	+ Adversarial training (%)	Detection failure rate (%)
**FGSM**	ϵ = 0.30	99.51	77.03	88.41	22.48
	ϵ = 0.50	99.51	42.72	65.34	56.79
**PGD (Iter = 10**, ***L*_∞_)**	ϵ = 0.30	99.51	95.71	97.12	3.80
	ϵ = 0.50	99.51	59.48	78.65	40.03
CW (*L*_2_ Norm)	*c* = 2 × 10^−3^, κ = 0.2	99.87	88.64	92.57	11.23

Under adversarial conditions, the model performance decreases as perturbation strength increases. For instance, under FGSM with ϵ = 0.50, the accuracy drops to 42.72%, while PGD with ϵ = 0.50 results in an accuracy of 59.48%. For the CW-L2 attack, the model achieves an accuracy of 88.64%, indicating relatively lower degradation under this attack. To further enhance robustness, adversarial training is incorporated, resulting in significant performance improvements across all attack types. For example, accuracy under FGSM (ϵ= 0.50) improves to 65.34%, and under PGD (ϵ = 0.50) to 78.65%, while CW-L2 accuracy increases to 92.57%. These results demonstrate that adversarial training effectively strengthens the model's resistance to adversarial perturbations without compromising baseline performance. The robustness trends across varying perturbation levels are illustrated in Figure 9, highlighting the stability and suitability of the proposed model for security-sensitive applications.

**Figure 9:**
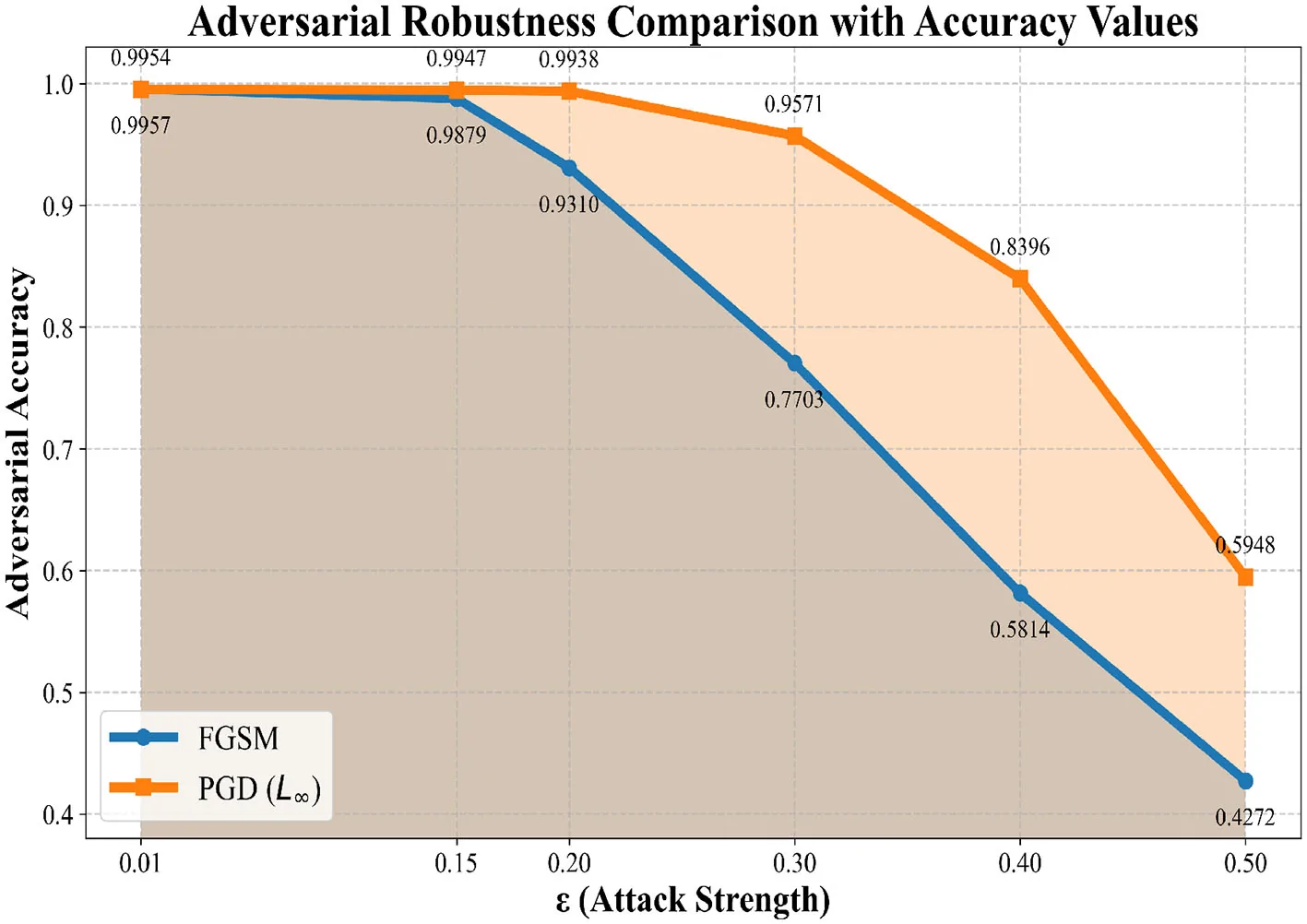
Adversarial robustness under FGSM and PGD attacks at varying perturbation strengths (ϵ), showing accuracy degradation and recovery with adversarial training.

### 4.9 Federated learning integration: results and discussion

Federated Learning (FL) is incorporated in the proposed work to enable privacy-preserving behavioral biometric authentication (Zhang et al., 2021; Wen et al., 2023). In this approach, multiple client devices collaboratively train a global model without sharing sensitive data such as keystroke or mouse dynamics, thereby preserving user privacy. The federated setup is evaluated with multiple client configurations (5, 10, and 20 clients) to better reflect realistic deployment scenarios, where each client represents an individual user device. Data preprocessing, feature engineering, and local SMOTE-based class balancing are performed on each client, followed by local training of the proposed Hybrid Transformer-CNN model. To further enhance privacy, Differential Privacy (DP) is incorporated during local training through gradient clipping (C = 1.0) and the addition of Gaussian noise with varying noise multipliers. The corresponding privacy budgets (ϵ) are reported, with δ fixed at 1 × 10^−5^, ensuring a formal privacy guarantee. Only privacy-preserving model updates are transmitted to the central server, where they are aggregated using the Federated Averaging (FedAvg) algorithm, and the updated global model is redistributed to clients over multiple communication rounds, as illustrated in Figure 10.

**Figure 10:**
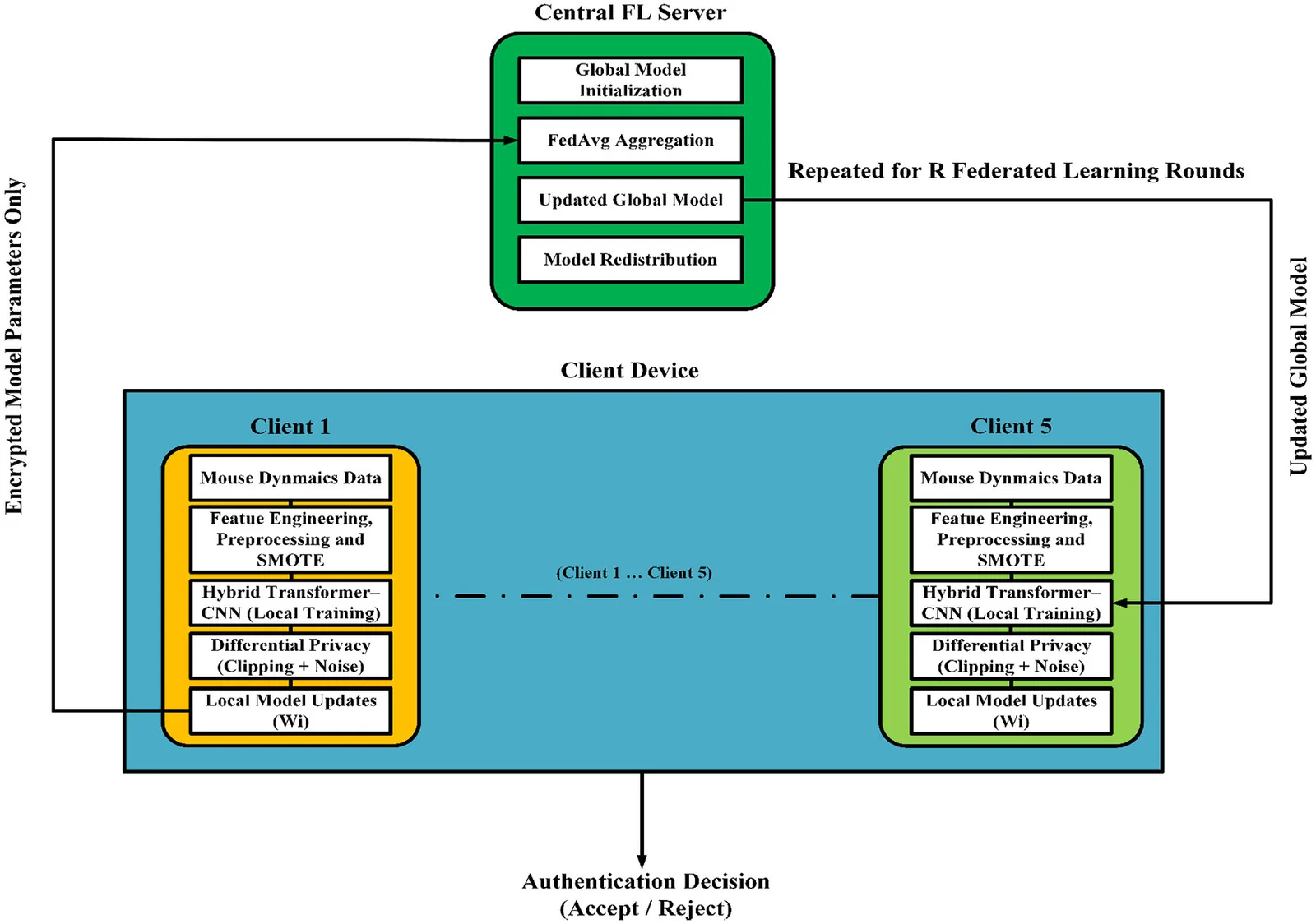
Federated learning-based behavioral biometric authentication architecture.

The federated model was trained for 25 communication rounds, with each client performing three local epochs using a batch size of 64 before transmitting its update to the server. The present implementation follows synchronous aggregation, where all configured clients participate in each communication round. In practical deployments, client availability may vary because of network interruptions, device inactivity, limited battery capacity, or communication failures, which may result in temporary client dropouts and fewer updates per round. Such conditions can influence convergence speed and communication efficiency. The current study does not explicitly simulate asynchronous communication or dynamic client participation; therefore, evaluating client dropout, communication failures, and asynchronous or staleness-aware aggregation is considered an important direction for future work.

The performance of the proposed framework is evaluated through both numerical results and visual analysis. Table 10 presents the impact of varying noise levels, number of clients, and privacy parameters on model accuracy, along with communication costs, highlighting the trade-off between privacy and performance. The results show that increasing noise levels improves privacy but reduces accuracy, while increasing the number of clients leads to higher communication overhead. In addition, a comparison with a robust aggregation method (Trimmed Mean with Secure Aggregation) is included, demonstrating improved resilience under certain configurations. To evaluate the impact of non-IID data distributions, Dirichlet-based partitioning is employed with varying concentration parameters (α = 0.1, 0.5, 1.0). The degree of heterogeneity is quantified using KL divergence, ranging from 1.0769 (highly non-IID) to 0.2980 (near-IID). As shown in Table 11, the proposed model maintains consistent performance across all settings, demonstrating robustness under varying data distributions. To analyze the impact of federated learning and differential privacy, a comparison is made between centralized training, FL without DP, and FL with DP. The centralized model achieves the highest accuracy (99.85%), followed by FL without DP (99.72%), while FL with DP (σ = 0.05) achieves 98.50%. These results highlight the trade-off between privacy and performance, showing that federated learning maintains competitive accuracy, with a moderate reduction when differential privacy is applied.

**Table 10:** Federated learning performance under differential privacy with varying clients, noise levels, and aggregation methods.

Clients	Noise (σ)	FedAvg + DP	Trimmed mean + DP + Secure Agg.	Comm. (MB)	ϵ	δ	C
5	0.05	0.969	**0.9678**	265.24	5.00	1 × 10^−5^	1.0
5	0.10	0.785	0.8019	265.24	2.50	1 × 10^−5^	1.0
5	0.20	0.522	0.5006	265.24	1.25	1 × 10^−5^	1.0
10	0.05	0.770	0.8105	530.48	5.00	1 × 10^−5^	1.0
10	0.10	0.539	0.5552	530.48	2.50	1 × 10^−5^	1.0
10	0.20	0.392	0.3313	530.48	1.25	1 × 10^−5^	1.0
20	0.05	0.529	0.5385	1060.95	5.00	1 × 10^−5^	1.0
20	0.10	0.399	0.3941	1060.95	2.50	1 × 10^−5^	1.0
20	0.20	0.223	0.2351	1060.95	1.25	1 × 10^−5^	1.0

Bold values indicate the highest accuracy achieved by each aggregation method.

**Table 11:** Impact of non-IID data distribution on model performance using dirichlet partitioning.

α (Dirichlet)	KL divergence	Accuracy (%)
0.1	1.0769	97.55
0.5	0.5086	99.83
1.0	**0.2980**	**99.87**

Bold values indicate the lowest KL divergence and highest authentication accuracy.

The FL loss and accuracy curves, depicted in Figure 11a, reveal the global model's stable convergence despite decentralized and privacy-preserving training. Figure 11b displays the fairness and robustness outcomes, which point to user-balanced learning and resistance to noisy, non-IID client data. The analysis of client contribution (Figure 12) takes it a step further by showing that all clients are quite significant in the training process, with no single client being the dominant one. All in all, these findings give assurance that the alliance of federated learning with the proposed model leads to a very accurate, fair, and privacy-preserving behavioral biometric authentication system fit for real-world deployment.

**Figure 11:**
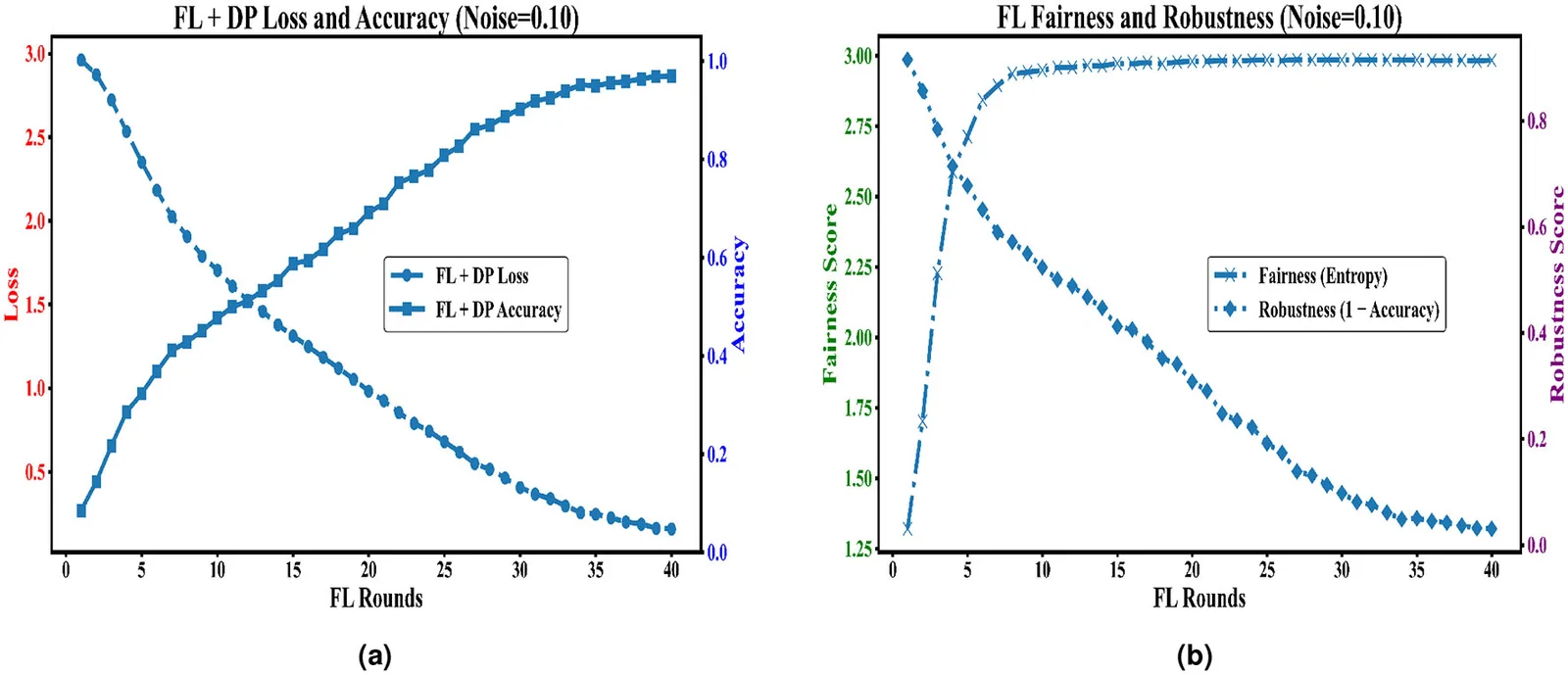
Federated learning performance under differential privacy with noise multiplier σ = 0.10: **(a)** global loss and accuracy convergence across 25 communication rounds; **(b)** fairness and robustness trends across participating clients.

**Figure 12:**
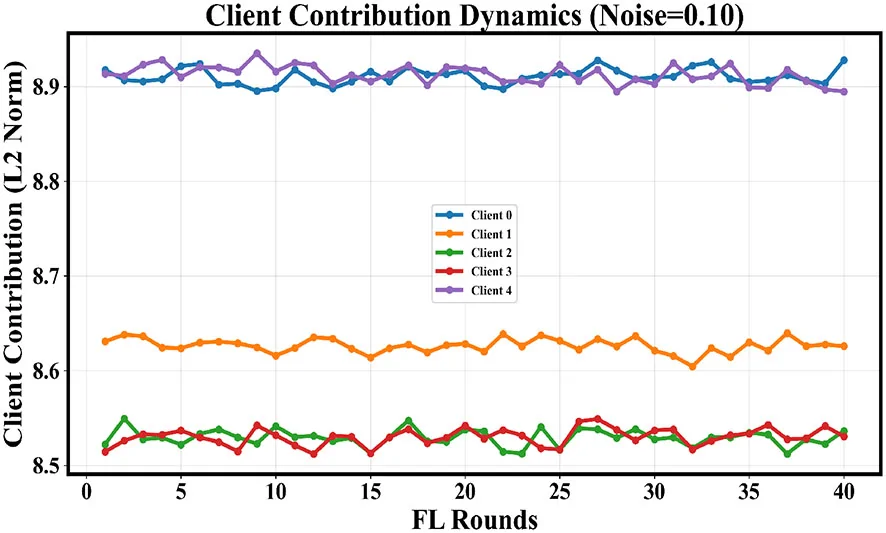
Client contribution dynamics across 25 federated learning rounds with differential privacy (σ = 0.10), showing balanced participation across clients.

### 4.10 Spoofing attack evaluation using GAN

To evaluate the robustness of the proposed model against spoofing attacks, a Generative Adversarial Network (GAN) is employed to generate synthetic behavioral samples that mimic legitimate user patterns. The GAN is trained for 300 epochs using a generator-discriminator framework, where the discriminator loss stabilizes around 1.17 and the generator loss around 0.80, indicating stable adversarial training. The generated samples capture realistic temporal patterns in mouse dynamics and are used to simulate structured spoofing attacks.

The adversarial samples are then used to evaluate the model's ability to distinguish between genuine and spoofed inputs. The results, presented in Table 12, show that the proposed model achieves a GAN-based spoof detection rate of 80.28%, indicating its capability in identifying structured adversarial patterns. The model maintains a clean accuracy of 99.53%, indicating that robustness enhancements do not degrade normal performance. In addition, random noise perturbations are applied to the input features to simulate unstructured attacks, where a perturbation factor is introduced to alter feature values. Under this setting, the detection rate decreases to 52.81%, highlighting the increased difficulty in detecting such perturbations. These results indicate that the proposed framework is effective against structured spoofing attacks generated using GANs, while further improvements are required to enhance robustness against generalized perturbations.

**Table 12:** Spoofing attack evaluation results.

Metric	Value
Clean accuracy	0.9953
GAN detection rate	0.8028
Noise detection rate	0.5281

## 5 Conclusion

The use of behavioral biometrics in continuous authentication has been recognized as an effective approach to overcome the limitations of conventional password-based systems. Mouse dynamics enable continuous and non-intrusive user verification during normal system interaction (Liang et al., 2020). This study proposes a Hybrid Transformer-CNN architecture integrated with federated learning and differential privacy to achieve secure and privacy-preserving authentication, together with robustness against adversarial attacks. Experimental results demonstrate the effectiveness of the proposed framework, achieving an authentication accuracy of 99.51%, precision of 99.53%, recall of 99.51%, and F1-score of 99.51%, along with a low false positive rate of 0.000256 and equal error rate of 0.000068. Compared with the baseline models, the improved performance can be attributed to the complementary learning capabilities of the Transformer and CNN, where the Transformer captures longer-range behavioral dependencies and the CNN extracts local movement patterns. Engineered mouse-dynamics features such as movement speed, acceleration, jerk, idle time, straightness, angle change, and direction changes provide complementary temporal, dynamic, and geometric information for user discrimination. Adversarial training improves robustness by exposing the model to perturbed samples and reducing its sensitivity to malicious input variations. Federated learning maintains competitive performance by aggregating locally learned behavioral information without transferring raw mouse-dynamics data, while differential privacy introduces the expected privacy-utility trade-off. The system also achieves real-time detection with a latency of 0.000037 seconds per sample.

The robustness evaluation indicates that the model maintains performance under adversarial attacks, with accuracies of 42.72% (FGSM), 59.48% (PGD), and 88.64% (CW-L2) at higher perturbation levels. With adversarial training, the robustness further improves, achieving 65.34% (FGSM), 78.65% (PGD), and 92.57% (CW-L2), demonstrating enhanced resistance to adversarial perturbations. Furthermore, federated learning experiments demonstrate a favorable privacy-utility trade-off, achieving 96.73% accuracy at a noise level of σ = 0.10. Under non-IID data distributions, the model achieves 97.55% accuracy at α = 0.1 and up to 99.87% at α = 1.0, confirming robustness across varying data heterogeneity. The open-set evaluation further shows strong authentication performance, with average FAR, FRR, and EER values of 0.00066, 0.00084, and 0.00066, respectively, along with effective performance under per-user threshold settings. In summary, the proposed framework achieves high authentication accuracy with robust performance and privacy preservation. Future work will focus on large-scale deployment, cross-device authentication, and adaptive learning to address behavioral drift. Although session-disjoint cross-validation and open-set evaluation were conducted, strict chronological evaluation using earlier sessions for training and later sessions for testing remains an important direction for future research. Such an evaluation would provide further evidence of the model's robustness to temporal changes in user behavior.

## Data Availability

Publicly available datasets were analyzed in this study. This data can be found here: The dataset is available in the public repository as follows: https://www.kaggle.com/datasets/jaafarnejm/mouse-dynamics-for-user-authentication.
